# Analysis of Microorganism Diversity in *Haemaphysalis longicornis* From Shaanxi, China, Based on Metagenomic Sequencing

**DOI:** 10.3389/fgene.2021.723773

**Published:** 2021-09-09

**Authors:** Runlai Cao, Qiaoyun Ren, Jin Luo, Zhancheng Tian, Wenge Liu, Bo Zhao, Jing Li, Peiwen Diao, Yangchun Tan, Xiaofei Qiu, Gaofeng Zhang, Qilin Wang, Guiquan Guan, Jianxun Luo, Hong Yin, Guangyuan Liu

**Affiliations:** ^1^State Key Laboratory of Veterinary Etiological Biology, Key Laboratory of Veterinary Parasitology of Gansu Province, Lanzhou Veterinary Research Institute, Chinese Academy of Agricultural Sciences, Lanzhou, China; ^2^Gansu Agriculture Technology College, Lanzhou, China; ^3^Animal Disease Prevention and Control Center of Qinghai Province, Xining, China; ^4^Jiangsu Co-innovation Center for the Prevention and Control of Important Animal Infectious Disease and Zoonose, Yangzhou University, Yangzhou, China

**Keywords:** metagenomic, *Haemaphysalis longicornis*, microorganism diversity, tick-borne pathogens, symbiotic microorganisms

## Abstract

Ticks are dangerous ectoparasites of humans and animals, as they are important disease vectors and serve as hosts for various microorganisms (including a variety of pathogenic microorganisms). Diverse microbial populations coexist within the tick body. Metagenomic next-generation sequencing (mNGS) has been suggested to be useful for rapidly and accurately obtaining microorganism abundance and diversity data. In this study, we performed mNGS to analyze the microbial diversity of *Haemaphysalis longicornis* from Baoji, Shaanxi, China, with the Illumina HiSeq platform. We identified 189 microbial genera (and 284 species) from ticks in the region; the identified taxa included *Anaplasma* spp., *Rickettsia* spp., *Ehrlichia* spp., and other important tick-borne pathogens at the genus level as well as symbiotic microorganisms such as *Wolbachia* spp., and *Candidatus* Entotheonella. The results of this study provide insights into possible tick-borne diseases and reveal new tick-borne pathogens in this region. Additionally, valuable information for the biological control of ticks is provided. In conclusion, this study provides reference data for guiding the development of prevention and control strategies targeting ticks and tick-borne diseases in the region, which can improve the effectiveness of tick and tick-borne disease control.

## Introduction

Ticks are important disease vectors that serve as hosts for various microorganisms; they can transmit etiological agents, including bacteria, viruses, and parasitic protozoa (Sanchez-Vicente et al., 2019; Velay et al., 2019), which cause a variety of animal diseases, including zoonoses transferred from animal to animal or human (Sharifah et al., 2020). In several countries, tick-borne diseases have been reported to cause immeasurable economic losses and negatively impact livestock development (Yang et al., 2018; Sahara et al., 2019; Zeb et al., 2020). Ticks are blood-sucking parasites that can cause host mortality *via* mechanisms (Edlow and McGillicuddy, 2008) such as anemia and tick paralysis. Therefore, analysis of tick microbial diversity is very important for identifying unknown pathogens or detecting known pathogens early and is beneficial for determining new biological control methods based on symbiotic microorganisms.

*Haemaphysalis longicornis* is a dominant tick species in China (Jia et al., 2020) that is prevalent in multifarious climatic environments in the northern and southern regions of China. It is an important pathogen vector and has one of the largest pathogen loads in China (Zhao et al., 2021); it can harbor pathogens such as *Anaplasma* spp., *Rickettsia* spp., and severe fever with thrombocytopenia syndrome virus (Luo et al., 2015; Jiang et al., 2018; Qin et al., 2018). Moreover, multiple human infections of tick-borne pathogens have been reported to be due to bites by *H. longicornis* (Kondo et al., 2017; Li et al., 2018, 2020), so this tick has become a focus of public health. Baoji City is an animal husbandry region in China with abundant wildlife resources (He, 2012). *H. longicornis*, as a pathogen vector, may cause epidemics due to transmission between livestock and wildlife in the region; therefore, understanding tick-borne pathogens transmitted by *H. longicornis* is significant for the protection of wildlife and livestock in the region.

Metagenomic next-generation sequencing (mNGS) has been suggested to be useful for identifying total microbial species from a single sample. An advantage of the technique is that it can obtain abundant microorganism data rapidly and accurately, allowing the analysis of microorganism diversity. This technique has been widely applied in veterinary science and animal husbandry production (Minamoto et al., 2015; Patel et al., 2018; Mora-Diaz et al., 2020) because of this advantage. mNGS has also been widely used in the analysis of microorganism diversity in vectors (Lambert et al., 2019; Ravi et al., 2019), and mNGS is helpful for understanding insect-borne pathogens and guiding the development of control vectors by identifying new biocontrol agents among symbiotic microorganisms.

In this study, we used the mNGS technique to obtain data for DNA-seq and bioinformatic analyses and obtained microbial species information for *H. longicornis* collected from Baoji, Shaanxi Province. mNGS detected some important tick-borne zoonotic pathogens in the samples. Therefore, this study will be a useful resource for efforts aimed at tick-borne disease control and the biological control of ticks in this region.

## Materials and Methods

### Tick Collection and Preparation

Ticks were collected as adults from host animals (cattle) in Baoji, Shaanxi, China, and were partially fed. The ticks were collected on July 3, 2020. Tick collection was performed after obtaining permission from the farmer. The tick species were identified by morphological features according to the descriptions in the *Economic Insect Fauna of China* (Deng and Jiang, 1991). The ticks were stored at −20°C before DNA analysis. A total of 131 ticks were analyzed in this study.

### DNA Extraction

The ticks were placed into new 50-ml sterile centrifuge tubes. Then, after being washed once with 75% ethanol, the ticks were rinsed with normal saline until the liquid was clear. QIAamp^®^ DNA Mini Kit (Germany) was used to extract the total DNA from each tick according to the protocol of the manufacturer. Each DNA sample was stored at −20°C.

### Library Construction and DNA Sequencing

To ensure quality, reduce sequencing costs, and be as comprehensive as possible, we selected the best 50 samples randomly from all the DNA samples for sequencing and combined them in a 100-μl pooled sample that comprised 2 μl of DNA solution from each of the 50 samples. The pooled sample was transported in Drikold (at below 0°C) to Novogene in China. The pooled DNA sample was randomly fragmented into 350-bp fragments with a Covaris ultrasonic disruptor, and then the entire library was end-repaired, A-tailed, ligated with a full-length adaptor, and subjected to purification and PCR amplification at Novogene.

Clustering of the index-coded samples was performed on a cBot Cluster Generation System according to the instructions of the manufacturer. After cluster generation, DNA sequencing of 150-bp paired-end reads was conducted with the Illumina HiSeq platform at Novogene.

### Data Analysis

Low-quality data were excluded from the raw data to acquire clean data for subsequent analysis using Readfq (version 8)^1^ ; the obtained data were compared against tick DNA data using Bowtie2.2.4 software to filter out host reads (Karlsson et al., 2012, 2013), and the parameters were as follows: -end-to-end, -sensitive, -I 200, and -X 400. Then, metagenome assembly, gene prediction, abundance analysis, and taxonomy prediction were completed according to the information analysis of the Metagenomic Project of Novogene Content. The detailed methods were as follows: For metagenome assembly, the clean data were assembled and analyzed (Luo et al., 2012) with SOAPdenovo software (V2.04),^2^ and the parameters (Scher et al., 2013; Qin et al., 2014; Brum et al., 2015; Feng et al., 2015) were as follows: -d 1, -M 3, -R, -u, -F, and -K 55. Then, the assembled scaffolds were interrupted at N positions to produce scaffolds without Ns (Mende et al., 2012; Nielsen et al., 2014; Qin et al., 2014), called scaftigs (i.e., continuous sequences within scaffolds), using SOAPdenovo (V2.04). Fragments shorter than 500 bp were filtered from all scaftigs for statistical analysis (Li et al., 2014; Qin et al., 2014; Zeller et al., 2014; Sunagawa et al., 2015).

For gene prediction and abundance analysis, scaftigs (≥ 500 bp) assembled from the sample were used for the open reading frame (ORF) (Zhu et al., 2010; Karlsson et al., 2012, 2013; Mende et al., 2012; Nielsen et al., 2014; Oh et al., 2014) prediction by MetaGeneMark software (V2.10),^3^ and sequences shorter than 100 nt (Qin et al., 2010, 2014; Li et al., 2014; Nielsen et al., 2014; Zeller et al., 2014) were filtered from the prediction results with the default parameters. For ORF prediction, CD-HIT (Li and Godzik, 2006; Fu et al., 2012) software (V4.5.8)^4^ was adopted to eliminate redundancy and obtain the unique initial gene catalog with the parameter options (Zeller et al., 2014; Sunagawa et al., 2015) -c 0.95, -G 0, -aS 0.9, -g 1, and -d 0. The clean data of the sample were mapped to the initial gene catalog using Bowtie 2.2.4 with the parameter settings (Li et al., 2014; Qin et al., 2014) -end-to-end, -sensitive, -I 200, and -X 400, and the number of reads in the sample mapping to individual genes was determined. The genes with less than or equal to two reads (Qin et al., 2012; Li et al., 2014) in the sample were filtered out to obtain the gene catalog (unigenes) used for subsequent analysis.

For taxonomy prediction, DIAMOND (Buchfink et al., 2015) software (V0.9.9)^5^ was used to blast the unigenes to the sequences of bacteria, eukaryote, archaea, and viruses, which were all extracted from the nucleotide (NR) database (version 2018-01-02)^6^ of NCBI with the parameter settings blastp and -e 1e-5. To obtain the final alignment results of each sequence, as each sequence may have multiple alignment results, the results with *e-*values less than or equal to the smallest *e*-value × 10 (Oh et al., 2014) were selected. Taxon classification with MEGAN (Huson et al., 2011) software using the last common ancestor (LCA) algorithm (Lowest_common_ancestor)^7^ was performed to confirm the species annotation information of the sequences. A table of the number of genes and the abundance information of the sample for each taxonomic level (kingdom, phylum, class, order, family, genus, and species) was produced based on the LCA annotation results and the gene abundance table. Krona analysis (Ondov et al., 2011) was used to visualize the results of the species annotation.

## Results

### Tick Identification

In total, 131 tick samples were collected from Baoji, Shaanxi Province, in July 2020. The samples were identified as *H. longicornis* (Acari: Ixodidae).

### Overview of DNA Sequencing

**FIGURE 1:**
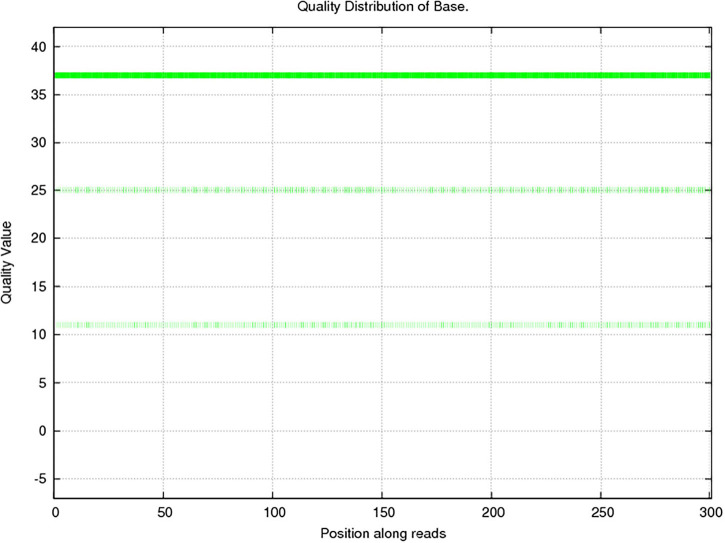
Sequencing quality distribution of bases. The quality of the sequencing data was mainly distributed above Q20, which guarantees the normal conduct of subsequent high-level analyses. The abscissa presents the position on the reads, and the ordinate presents the base mass distribution over the position on the reads.

**FIGURE 2:**
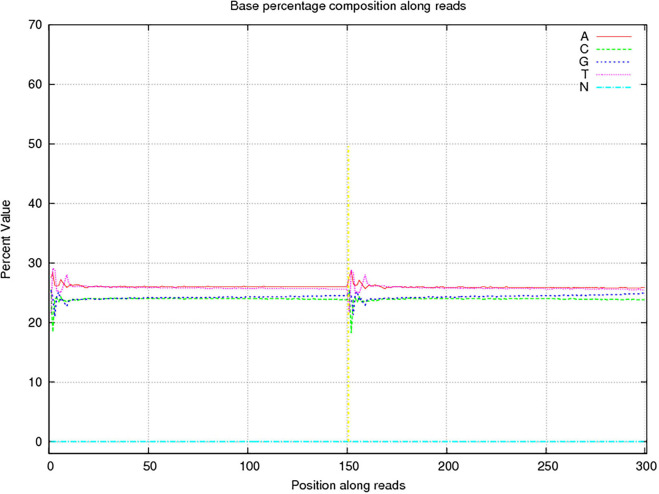
Sequencing base percentage composition among reads. The abscissa presents the position on the reads, the ordinate presents the content of a certain base at that position, and the five colors indicate the proportions of content of the four bases of ATGC and undetected bases (Ns).

**FIGURE 3:**
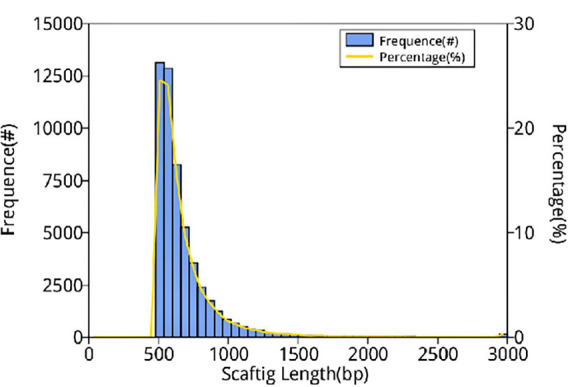
Distribution of scaftig lengths across samples. The first vertical axis (frequency, #) indicates the number of scaftigs, the second vertical axis (percentage, %) indicates the percentage of the number of scaftigs, and the horizontal axis indicates the scaftig length.

**FIGURE 4:**
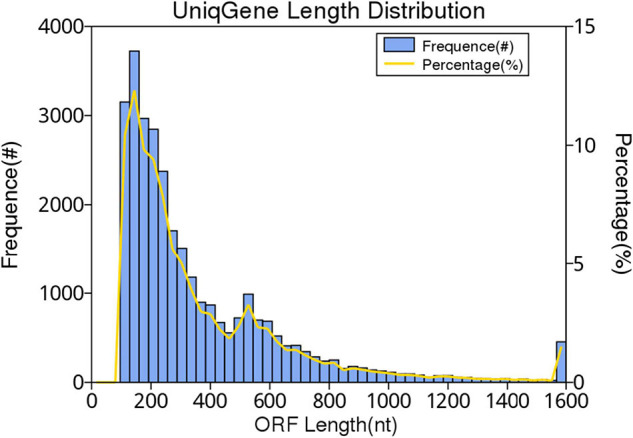
Gene catalog length distribution. The first vertical axis frequency (#) indicates the number of genes in the catalog, the second vertical axis percentage (%) represents the percentage of the number of genes in the catalog, and the horizontal axis indicates the length of the gene in the gene catalog.

In this study, 10,117.72 Mbp of clean data were generated by sequencing with the Illumina HiSeq platform; the effective data rate was 99.64%, and the results of the quality control are shown in Figures 1, 2. After a single sample assembly, 58,543,947-bp scaffolds were obtained. Then, a total of 37,959,124-bp scaftigs were obtained by interrupting scaffolds at the N-site; the distribution of scaftig lengths across samples is shown in Figure 3. After obtaining the assembly results, MetaGeneMark software was used for gene prediction, basic gene catalog information statistics were obtained (Table 1 and Figure 4), and a total of 30,341 ORFs were identified. After excluding redundant sequences, 30,255 ORFs were identified, and among them, the number of complete genes was 9,458, accounting for 31.26% of the ORFs.

**TABLE 1:** Gene catalog and basic information.

Catalog	Amount
ORFs NO.	30,255
Integrity:all	9,458 (31.26%)
Integrity:end	9,558 (31.59%)
Integrity:none	2,601 (8.6%)
Integrity:start	8,638 (28.55%)
Total Len. (Mbp)	11.42
Average Len. (bp)	377.51
GC percent	50.74

*ORF No. indicates the number of genes in the gene catalog; integrity:start indicates the number and percentage of genes containing only the start codon; integrity:end indicates the number and percentage of genes containing only stop codons; integrity:none indicates the number and percentage of genes with no start or stop codons; integrity:all indicates the percentage of the number of complete genes (both start and stop codons); Total len. (Mbp) represents the total length of a gene in a gene catalog in millions; Average len. indicates the average length of the gene in the gene catalog; GC percent represents the overall GC content value of a gene in the predicted gene catalog.*

### Taxonomy Prediction and Diversity Analysis

**FIGURE 5:**
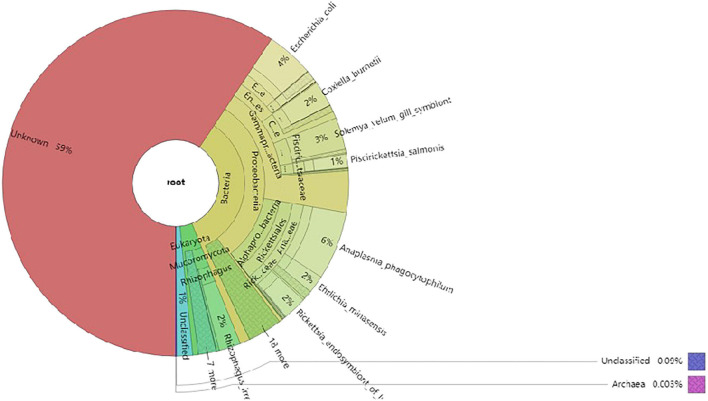
The Krona diagram of taxonomy prediction. In the figure, circles represent different classification levels (kingdom, phylum, class, order, family, and genus) from inside to outside. The size of the sector represents the relative abundance of different species.

The non-redundant gene catalog (unigenes) was BLASTP-aligned with the NR database of NCBI (see text footnote 6; version: 2018-01-02) using DIAMOND software. Based on the LCA algorithm, species annotation was performed. There were 30,255 genes after the initial redundancy removal, and the number of ORFs that were annotated to the NR database was 7,754 (25.63%). The proportion of ORFs annotated to the genus level was 67.08% (189 genera) and that annotated to the species level was 58.72% (284 species). According to the results, the most common species of microorganisms was bacteria; a total of 145 species were identified. The next was eukaryote and had 116 species. The last was virus and archaea; there were 21 species and two species, respectively. The result of the species annotation is shown in the Krona figure (taxonomy.krona.html, Figure 5).

**FIGURE 6:**
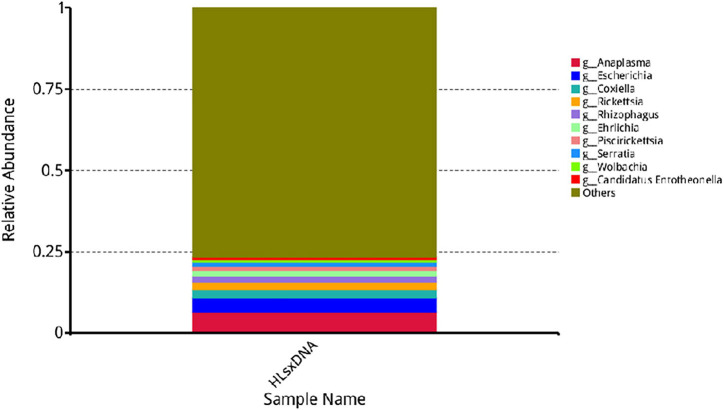
Relative abundance of the top 10 genera. The horizontal axis presents the sample names, the vertical axis presents the relative proportion of species annotated to a certain type, and the genera corresponding to individual color blocks are indicated in the legend on the right.

**FIGURE 7:**
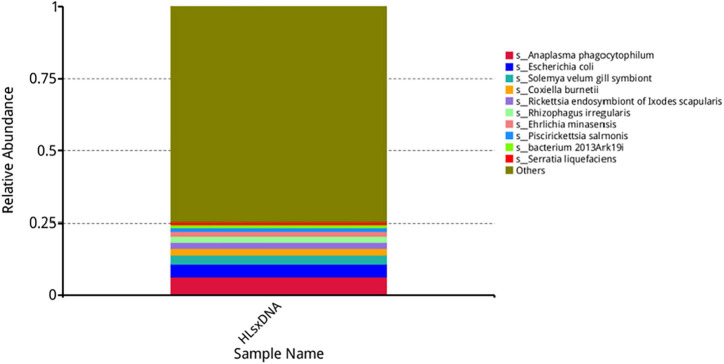
Top 10 most abundant species. The horizontal axis presents the sample names, the vertical axis presents the relative proportion of species annotated to a certain type, and the species corresponding to individual color blocks are indicated in the legend on the right.

*Anaplasma*, *Escherichia*, *Coxiella*, *Rickettsia*, *Rhizophagus*, *Ehrlichia*, *Piscirickettsia*, *Serratia*, and *Wolbachia* were the predominant genera based on relative abundance in all microorganisms. *Anaplasma phagocytophilum*, *Escherichia coli*, *Solemya velum* gill symbiont, *Coxiella burnetiid*, *Rickettsia endosymbiont* of *Ixodes scapularis*, *Rhizophagus irregularis*, *Ehrlichia minasensis*, *Piscirickettsia salmonis*, and bacterium 2013Ark19i were the predominant species based on relative abundance in all microorganisms. The predominant genera and species are shown in Figures 6, 7, respectively.

The predominant eukaryotes were *R. irregularis*, *Metarhizium anisopliae*, *Enterospora canceri*, *Rhizopus delemar*, *Rhizoctonia solani*, *Absidia glauca*, *Puccinia striiformis*, *Smittium culicis*, and *Nosema apis* on relative abundance in eukaryote. In addition, *Lymphocystis disease virus Sa*, *Cotesia sesamiae bracovirus*, and *Autographa californica multiple nucleopolyhedrovirus* were the predominant viruses on relative abundance in virus, and the only two archaea were *Thermococcus* sp. 2319 × 1 and *Lokiarchaeum* sp. GC14_75. Those results are shown in the Krona figure (taxonomy.krona.html, Figure 5).

## Discussion

Ticks are important pathogen vectors (Sharifah et al., 2020). In this study, *H. longicornis* was collected from the Qinling area in Baoji, Shaanxi, China. Then, tick DNA was extracted and analyzed with mNGS technology. The primary objective was to understand the microbial diversity in *H. longicornis* in the region. Prior to sequencing, 50 samples of the 131 collected samples were pooled. Finally, a total of 284 microorganisms were annotated and classified as bacteria, eukaryote, viruses, or archaea. Common pathogens transmitted by *H. longicornis*, such as *Rickettsia* spp., *Anaplasma* spp., and *Ehrlichia* spp., were detected. Most known *Rickettsia* species belong to the spotted fever group (Abdad et al., 2018) and cause spotted fever. *Anaplasma* and *Ehrlichia* belong to Anaplasmataceae and are prevalent and potentially fatal arthropod-borne pathogens (Dumler, 2005). Previous studies on *H. longicornis*-borne pathogens have revealed that these pathogens are commonly detected and widely distributed in *H. longicornis* in all regions of China. The *Rickettsia* positivity rate was 7.36% in Liaoning, China (Xu et al., 2019), and the *Anaplasma* and *Ehrlichia* positivity rates were 2.2 and 0.8%, respectively, in northeastern China (Wei et al., 2016). The rate of *Ehrlichia* positivity was 1.82%, and the rate of *Anaplasma* positivity was 11.82% in Zhejiang, China (Hou et al., 2019). Moreover, *Rickettsia* (0.67%, 2/298), *Anaplasma* (3.02%, 9/298), and *Ehrlichia* (1.01%, 3/298) were detected in central China (Chen et al., 2014). The results of this study show that these pathogens may be common in the region and that the incidence of diseases caused by these pathogens could be very high; therefore, these diseases need to be monitored to ensure successful animal husbandry and healthy production in the region.

In addition to the abovementioned pathogens, *Coxiella* spp. (including *Coxiella burnetiid* and *Coxiella*-like endosymbiont) were detected, with a high relative abundance. *Francisella tularensis* was detected, but it had a low relative abundance in this study. Both *C. burnetii* and *F. tularensis* are important zoonotic pathogens (Maurin and Raoult, 1999; Proksova et al., 2019). The different abundances may be related to the carrying capacity of *H. longicornis*. Previous studies have found that *F. tularensis* did not persist in *H. longicornis* after artificial infection (Tully and Huntley, 2020). However, *Coxiella* has a symbiotic relationship with *H. longicornis*. Phylogenetic analysis has revealed that *C. burnetii* is closely related to *Coxiella*-like endosymbiont (Elsa et al., 2015; Trinachartvanit et al., 2018). As expected, these pathogens were detected in *H. longicornis* in this study, which suggests that epidemics of *Coxiella-*related diseases could occur in this area.

*Wolbachia* spp. was also detected in *H. longicornis*, with a high relative abundance in this study. Studies on *Wolbachia* in arthropods (Carvajal et al., 2019; Landmann, 2019) have confirmed that it is prevalent in mosquitoes and important for insect control (Beckmann et al., 2017; Madhav et al., 2020). However, only a few studies have reported the detection of *Wolbachia* genes in individual tick species, and none has reported the presence of *Wolbachia* in *H. longicornis* (Madhav et al., 2020). The results of this study suggest that *Wolbachia* is present in *H. longicornis*, but further investigation is required to determine whether it has the same cytoplasmic incompatibility function in *H. longicornis* as it does in other taxa.

In summary, this study using mNGS technology revealed the microbial species composition in *H. longicornis* in the Baoji Qinling region. Pathogenic infection *via* ticks is complex and diverse, and tick bites are a potential threat to wildlife, domestic animals, and people involved in forestry, livestock, and tourism in the region. Therefore, workers in relevant industries in the region need to adhere to surveillance measures and implement control strategies. It is very important to monitor for possible emerging tick-borne diseases by analyzing microbial species; monitoring may have profound and great implications for public health and the safety of animal husbandry in the region.

## Conclusion

In this study, microorganism diversity data were obtained from *H. longicornis* in Baoji, Shaanxi, China. The data indicated that pathogens harbored by *H. longicornis* may pose a great threat to animals and humans in this region. Therefore, it is important to control ticks in this region. In addition, the results provide evidence of the presence of *Wolbachia* spp., in *H. longicornis*. These results are of great significance for the prevention and control of ticks and tick-borne diseases in the region, as these data are necessary for guiding human and animal safety measures in the region.

## Data Availability Statement

The raw sequence data are available in the National Genomics Data Center (NGDC), the China National Center for Bioinformation (CNCB), using accession number CRA004680 (https://bigd.big.ac.cn/gsa/browse/CRA004680).

## Author Contributions

RC contributed to the methodology, formal analysis, and writing of the original draft. QR contributed to the methodology and validation. JLu, ZT, GG, JLu, and HY contributed to writing—review and editing. WL, BZ, and JLi contributed to formal analysis and writing of the original draft. PD, YT, and XQ contributed to formal analysis and resources. GZ and QW contributed to formal analysis. GL conceived and designed the experiment and contributed to writing—review and editing. All authors contributed to the article and approved the submitted version.

## Conflict of Interest

The authors declare that the research was conducted in the absence of any commercial or financial relationships that could be construed as a potential conflict of interest.

## Publisher’s Note

All claims expressed in this article are solely those of the authors and do not necessarily represent those of their affiliated organizations, or those of the publisher, the editors and the reviewers. Any product that may be evaluated in this article, or claim that may be made by its manufacturer, is not guaranteed or endorsed by the publisher.

## Footnotes

1
https://github.com/cjfields/readfq

2
http://soapdenovo2.sourceforge.net/

3
http://topaz.gatech.edu/GeneMark

4
http://www.bioinformatics.org/cd-hit

5
https://github.com/bbuchfink/diamond/

6
https://www.ncbi.nlm.nih.gov/

7
https://en.wikipedia.org/wiki/
